# Evaluating β Diversity as a Surrogate for Species Representation at Fine Scale

**DOI:** 10.1371/journal.pone.0151048

**Published:** 2016-03-04

**Authors:** Paul Beier, Fábio Albuquerque

**Affiliations:** 1 School of Forestry, Northern Arizona University, Flagstaff, Arizona, 86001, United States of America; 2 Science and Mathematics Faculty, Arizona State University, Mesa, AZ, 85212, United States of America; Clemson University, UNITED STATES

## Abstract

Species turnover or β diversity is a conceptually attractive surrogate for conservation planning. However, there has been only 1 attempt to determine how well sites selected to maximize β diversity represent species, and that test was done at a scale too coarse (2,500 km^2^ sites) to inform most conservation decisions. We used 8 plant datasets, 3 bird datasets, and 1 mammal dataset to evaluate whether sites selected to span β diversity will efficiently represent species at finer scale (sites sizes < 1 ha to 625 km^2^). We used ordinations to characterize dissimilarity in species assemblages (β diversity) among plots (inventory data) or among grid cells (atlas data). We then selected sites to maximize β diversity and used the Species Accumulation Index, SAI, to evaluate how efficiently the surrogate (selecting sites for maximum β diversity) represented species in the same taxon. Across all 12 datasets, sites selected for maximum β diversity represented species with a median efficiency of 24% (i.e., the surrogate was 24% more effective than random selection of sites), and an interquartile range of 4% to 41% efficiency. β diversity was a better surrogate for bird datasets than for plant datasets, and for atlas datasets with 10-km to 14-km grid cells than for atlas datasets with 25-km grid cells. We conclude that β diversity is more than a mere descriptor of how species are distributed on the landscape; in particular β diversity might be useful to maximize the complementarity of a set of sites. Because we tested only within-taxon surrogacy, our results do not prove that β diversity is useful for conservation planning. But our results do justify further investigation to identify the circumstances in which β diversity performs well, and to evaluate it as a cross-taxon surrogate.

## Introduction

Because many species have not been described and most species distributions have not been mapped, conservation planners have long used surrogates for conservation planning [1]. Surrogates are features that are well-mapped in the planning region, such as soil types, landforms, climate conditions, or occurrences of an easily-observed taxon. The idea is that a set of *p* sites selected to span diversity in the surrogate will efficiently represent diversity of species (or other biodiversity targets) whose true distributions are not known.

In this paper we evaluate β diversity as a surrogate, i.e., whether sites selected to span β diversity will efficiently represent species. We use the term β diversity as a synonym for species turnover or assemblage dissimilarity [2–7]. To use β diversity as a surrogate for species representation, we follow the “indirect environmental diversity” approach of Faith and Walker [3–6]. In this approach, sites (geographic places that could be selected for conservation) are arranged in an ordination in which distance between sites reflect dissimilarity of species assemblages of a well-mapped group (the indicator group). Then, given a budget sufficient to conserve *p* sites, a *p*-median selection algorithm selects sites to span the ordination. It is assumed that each assemblage reflects the responses of individual species to environmental variation among sites. Thus a site with an assemblage that differs from the assemblages at the previously selected sites is assumed to represent a new environmental setting, and the site is therefore predicted (on average) to represent additional species in the target group, even if it contains no new species in the indicator group. The original claim [4] was that sites spanning β diversity in an indicator taxon will efficiently represent targets in other taxa, but this claim obviously must apply with equal or greater force to targets in the indicator taxon [6]. Thus the use of β diversity as a surrogate entails the hypothesis that sites selected to span β diversity will efficiently represent species in the same taxon or another taxon.

A key assumption of the model is that each species has a unimodal distribution in environmental space. If this assumption is true, Faith and Walker [4] proved that sites selected to span ordination space will efficiently represent target species. This critical assumption could be false if species distributions were driven less by environmental conditions than by other factors, such as habitat degradation and loss due to human activities, species-specific dispersal limitations (e.g., after retreat of continental glaciers, or formation of a land bridge between land masses), stochastic colonization events, interspecific interactions, or frequent disturbance.

In this paper we test this critical assumption–and test β diversity as a surrogate–with 12 datasets, and assess whether the efficiency of β diversity varies with characteristics of the dataset such as spatial extent, spatial resolution (size of site), type of species data (inventory versus atlas), taxon, number of species, or stress value (badness of fit) of the ordination [8]. There has been only 1 previous attempt to determine how well sites selected to maximize β diversity represent species [6], and that test was done at a scale too coarse (2,500 km^2^ sites) to inform most conservation decisions. We extend this work to finer scales (sites < 1 ha to 625 km^2^).

For each of our 12 datasets, we used a single taxon as both the indicator taxon and the target taxon. We acknowledge that a conservation planner would want to use β diversity only as a cross-taxon surrogate, and that our within-taxon tests overestimate how well β diversity would represent biodiversity targets in another taxon. However, we could not find even a handful of datasets that describe occurrence of multiple taxa in cells no larger than 25 x 25 km. Despite the limitation of using within-taxon tests, our effort is the most comprehensive attempt to evaluate β-diversity as a surrogate for species representation. If β-diversity is a poor surrogate in tests using the same taxon as indicator and target, we would infer that the key assumption (species have unimodal distributions in environmental space) is false. On the other hand, if β diversity is a good within-taxon surrogate, then further investigation of β diversity as a cross-taxon surrogate would be warranted.

## Materials and Methods

To test whether β diversity is an efficient surrogate for within-taxon biodiversity, we analyzed 12 biological datasets. Each dataset consisted of a list of species present in each of > 200 sites. We selected datasets that attempted to document all species present, such that pseudo-absences probably indicated true absences in most cases. We further attempted to find datasets on different taxa, from different geographic regions, and to include both atlas and inventory datasets. Araujo et al. [6] suggested that β diversity might perform poorly at continental spatial extent (where common species may drive β diversity patterns) and for large (50x50 km) grain size (which cannot detect local gradients driven by restricted-range species). Accordingly, we selected datasets covering small to mid-sized regions, with grain sizes no larger than 25x25 km. We present results for all datasets analyzed. Five datasets were inventories of the focal taxon at all sites; 7 datasets were atlases summarizing occurrence records of the focal taxon (Table 1).

**Table 1:** Twelve datasets used to evaluate β diversity. In each “atlas” dataset, each site was a grid cell, survey efforts did not cover the entirety of each grid cell, and the sites collectively comprised the entire study area. In each “inventory” dataset, the sites were a systematic subsample of the study area.

Taxon and geographic area	Extent (km^2^)^a^	# Sites & Assemblages	Size of inventory site or grid cell	# Species	Type of dataset
Plants, Sequoya/Kings Canyon National Park, USA^b^	3497	545	<1 ha	854	Inventory
Plants, Sierra Nevada, Spain [9]	862	596	<1 ha	262	Inventory
Plants, Shenandoah National Park, USA^b^	810	351	<1 ha	728	Inventory
Plants, Chiapas, Mexico [10]	73,311	230	<1 ha	258	Inventory
Plants, UK [11]	243,610	2,242	100 km^2^	1,456	Atlas
Plants, Botswana ^c^	581,730	556	625 km^2^	2,237	Atlas
Plants, Namibia ^c^	825,615	998	625 km^2^	3,566	Atlas
Plants, Zimbabwe ^c^	390,757	360	625 km^2^	1,338	Atlas
Birds, Arizona, USA [12]	295,234	1,317	25 km^2^	359	Inventory
Birds, Spain [13]	505,992	5,301	100 km^2^	294	Atlas
Birds, Florida, USA [14]	170,304	1,028	196 km^2^ (7.5’)	211	Atlas
Mammals, Ireland ^d^	84,421	1,684	100 km^2^	39	Atlas

^a^ Approximate area within park or political boundary

^b^ Data from US National Park Service Inventory Products http://science.nature.nps.gov/im/inventory/veg/products.cfm (accessed 20 June 2014)

^c^ Data from South African National Biodiversity Institute, PRECIS accessed via http://www.gbif.org/dataset/1881d048-04f9-4bc2-b7c8-931d1659a354 on 2014-03-10

^d^ Data from the Atlas of Mammals in Ireland 2010–2015 dataset held by the National Biodiversity Data Centre www.biodiversityireland.ie, accessed via http://www.gbif.org/dataset/c585e6fb-fd76-426e-ae01-a32dc9de5689 on 2014-03-10

For each dataset, we created a matrix of the dissimilarity values (1 minus Jaccard similarity) between each pair of sites [7]. We chose Jaccard dissimilarity because this index is appropriate for presence-absence data, whereas Bray-Curtis and other dissimilarity metrics are appropriate for relative abundance data. Then we used nonmetric multidimensional scaling (NMDS [15]) and hybrid multidimensional scaling (HMDS [16]) to place each site in 2-dimensional space, where inter-site distance reflected β diversity (species turnover, dissimilarity between species assemblages). Within each ordination (NMDS or HMDS) we performed up to 1,000 iterations per random start and selected the ordination with the lowest stress value across 10 random starts. Matrix and ordination analyses were performed using R package *vegan* [17].

We created a regularly-spaced grid of 10,000 points across this ordination space and treated them as demand points in a *p*-median selection algorithm. This algorithm selects the set of *p* sites that minimizes the sum of distances (in ordination space) from each demand point to the nearest selected site. Specifically, we used the hybrid heuristic *p*-median procedure in POPSTAR [18] with 32 random starts, and hybridization among the top 10 solutions to identify a solution that best spans β-diversity space. In previous tests, including tests with pathological datasets (created with the sole purpose of being hard to solve), POPSTAR obtained solutions better than or within 0.1% of the best known solution [18]. We conducted 5 runs per dataset, varying *p* to select 15%, 20%, 25%, 30%, and 35% of the total number of sites in each dataset. We chose these levels to reflect performance of each surrogate at levels about as low as the current extent of the world’s protected areas (13% of land area [19]), increasing to various plausible levels of expansion of the protected area network [20]. These 5 sets of sites represented reserve networks selected to maximize β diversity across sites. We intersected the network with the species data (inventories or atlas) to calculate *S*, the number of species represented in each surrogate network.

**Fig 1:**
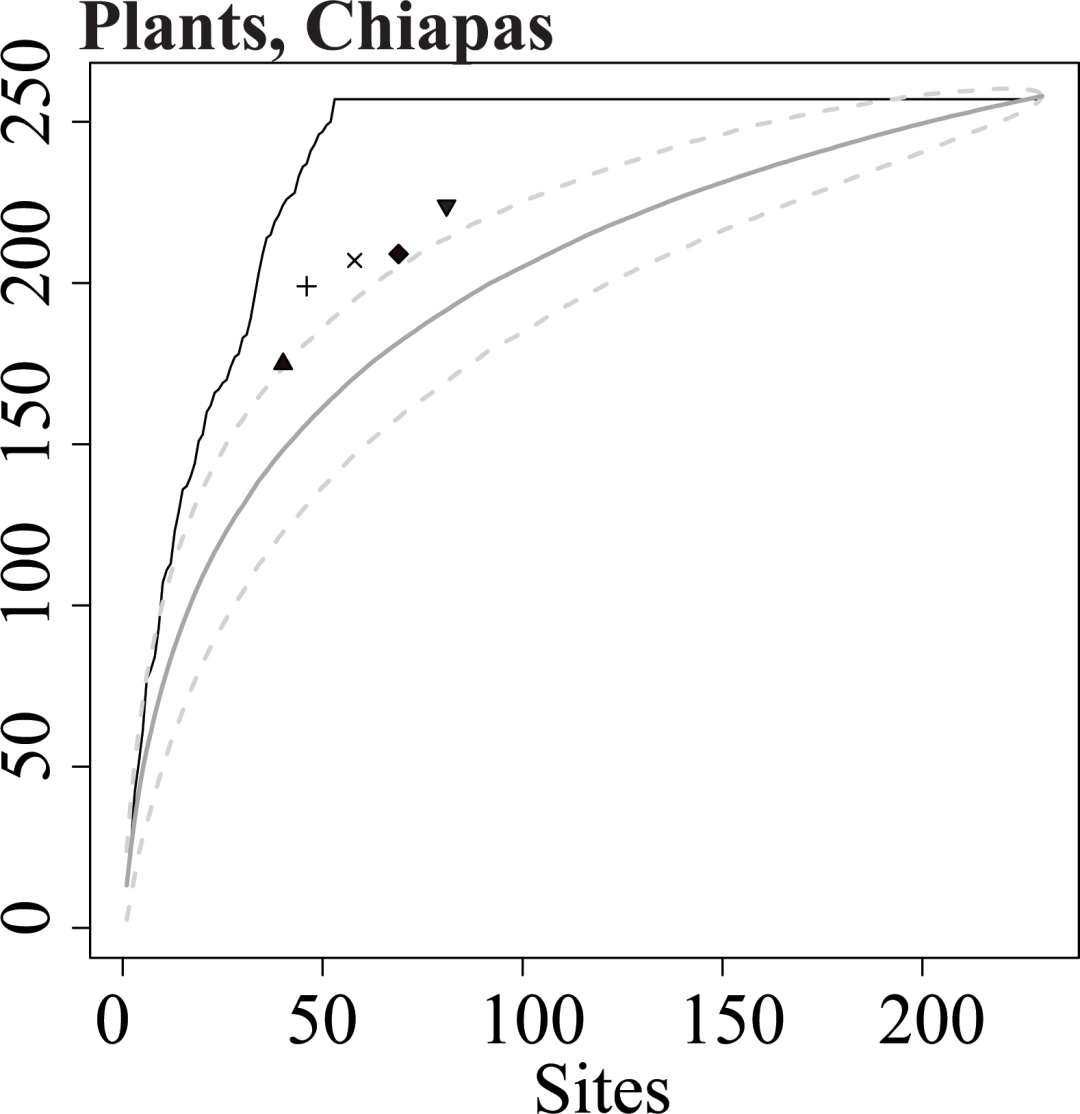
A full species accumulation curve for plants in Chiapas. The upper line (black) indicates the largest possible number of species represented at least once in a given number of sites, as estimated by Zonation [22]. The lower curve (gray) indicates the average number of species represented at least once in a randomly-selected set of sites; dashed lines enclose 95% of 1000 random sets. Symbols represent the number of species represented at least once in sites selected to maximize β diversity (dispersion of sites in a 2-dimensional ordination of species assemblages). From left to right, the symbols correspond to 15% (upward triangle), 20% (cross), 25% (x), 30% (diamond), and 35% (downward triangle) of all sites in the dataset. In Fig 2, data in the lower left portion of the graph are omitted to present the same results in higher resolution.

We used the Species Accumulation Index, SAI [1, 21] to evaluate the efficiency of the surrogate (selecting sites for maximum β diversity) in representing species within the taxon (Fig 1). SAI compares *S*, the number of species represented in the set of sites selected using the surrogate, to an optimum value *O* (the largest number of species that can be represented in the same number of sites) and to *R*, the mean number of species represented in the same number of randomly selected sites. We calculated *O* from the core area version of Zonation [22], a selection algorithm that starts with all sites tentatively “reserved” and progressively removes sites least needed to retain at least a small number (core) of occurrences of each species. To calculate *R*, we accumulated cells in random order and at each step we calculated the number of species represented at least once in the randomly selected cells. We repeated the random selection procedure 1,000 times, used the mean value as *R*, and calculated a 95% CI on *R*.

Formally, SAI = (*S-R*)/(*O-R*). SAI is scaled –∞ to 1; negative SAI indicates a worse than random result, 0 indicates random performance, and positive SAI is a measure of efficiency. For example, SAI of 0.6 indicates that the surrogate was 60% as effective as the optimal solution in its ability to improve on random selection of sites. We calculated SAI at 15%, 20%, 25%, 30%, and 35% of the landscape hypothetically reserved (Fig 1). We used the mean of these 5 SAI values as an overall estimate of surrogate performance for each dataset.

Across the 12 datasets, we used scatterplots, t-tests, and correlations to evaluate influence of size of study area, number of species, stress value of the ordination, taxon, size of grid cell (for atlas data), and type of biodiversity data (inventory, atlas) on SAI. After the counterintuitive finding that low stress values were associated with low SAI values, we examined ordination plots and Shepard plots, and experimented with deleting apparent outliers to try to discern patterns associated with poor performance.

## Results

**Fig 2:**
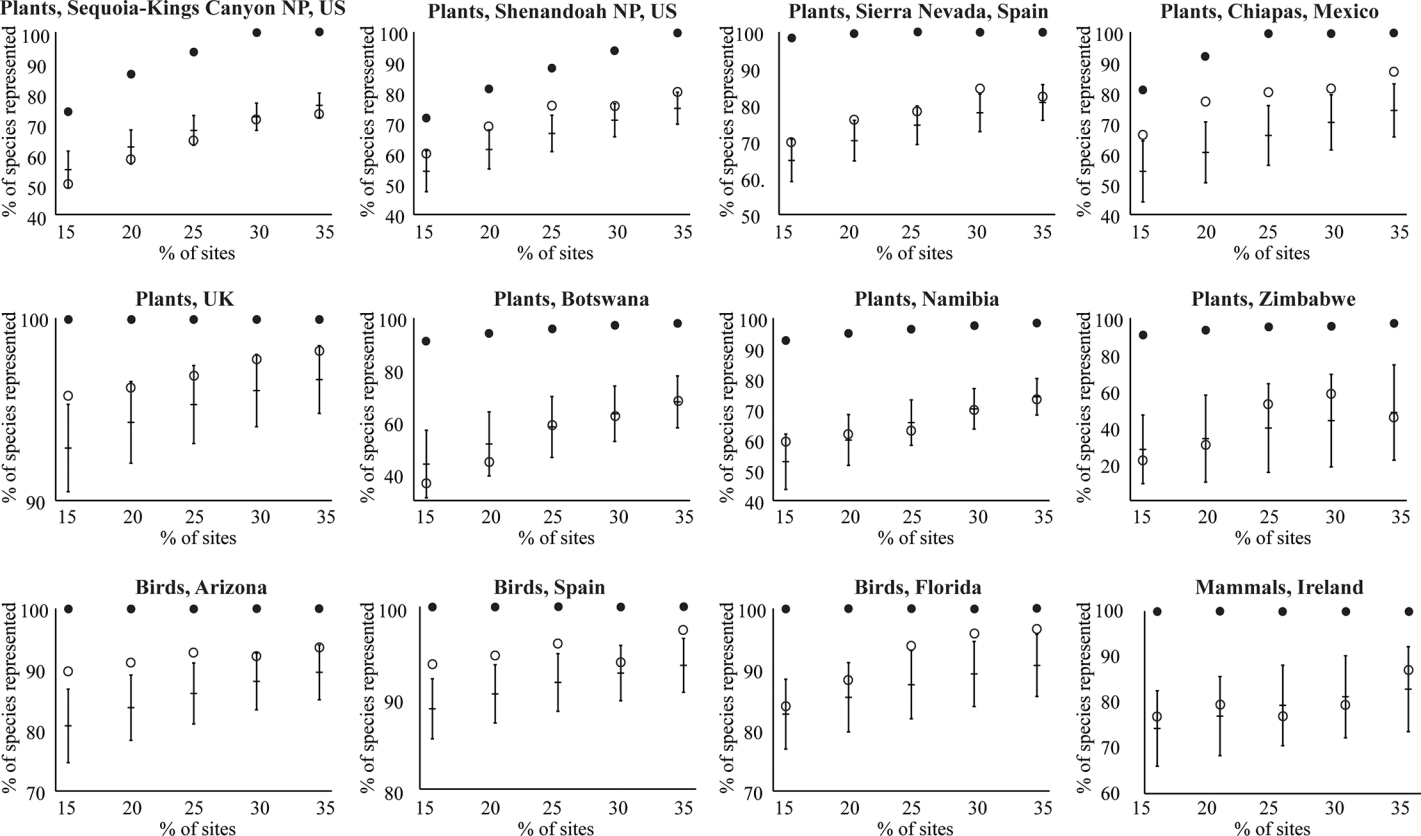
Efficiency of β diversity. Number of species represented at least once in sites selected to maximize β diversity (open circles) compared to the largest possible number of species represented at least once in the same number of sites (black circles, estimated by Zonation [21]), and the number of species represented at least once in the same number of randomly-selected sites (vertical bar indicates mean and 95% CI).

Across all 12 datasets, sites selected for maximum β diversity represented species at a median 25% of maximum efficiency. Average efficiencies (SAI) ranged from -15% to +45%, with an interquartile range of 4% to 41% (Table 2). The mean SAI of 21% was significantly greater than zero (one-sample t-test, 11 df, *P* < 0.0001). For 6 of the 12 datasets, sites selected for maximum β diversity represented species at 31% to 45% of maximum efficiency, with >30% efficiency at most percentages of the landscape (Table 2, Fig 2). These 6 datasets included birds in Arizona, Spain, and Florida, and plants in Chiapas (Mexico), United Kingdom, and Shenandoah National Park (USA). For plants in Sierra Nevada (Spain), β-diversity performed with average 18% efficiency, but only 1 of 5 SAI values was above the 95% CI for randomly selected sites, and efficiency was as low as 9% when 35% of the landscape was selected for the hypothetical conservation network. For the remaining five datasets, all SAI values were within the 95% confidence interval for the same number of randomly selected sites.

**Table 2:** Performance of β diversity in prioritizing sites for conservation, for each of 12 datasets. Performance (SAI) values indicate how efficiently β diversity represents species compared to the same number of randomly selected sites. Bold indicates values significantly above 95% CI of the same number of randomly-selected sites. Columns are arranged from highest to lowest mean SAI.

% of sites prioritized	Dataset											
	Plants, Chiapas	Birds, Arizona	Birds, Spain^a^	Plants, U.K.	Birds, Florida	Plants, Shenandoah NP^a^	Plants, Sierra Nevada (Spain) ^a^	Plants, Zimbabwe	Mammals, Ireland^a^	Plants, Namibia	Plants, Botswana	Plants, Sequoia-Kings Canyon NP
15%	**0.42**	**0.47**	**0.42**	**0.41**	0.07	0.33	0.15	-0.10	0.10	0.17	-0.16	-0.26
20%	**0.53**	**0.45**	**0.43**	0.33	0.19	**0.38**	0.20	-0.06	0.11	0.05	-0.16	-0.17
25%	**0.42**	**0.48**	**0.51**	0.34	**0.47**	**0.43**	0.15	0.23	-0.12	-0.08	0.01	-0.14
30%	**0.37**	0.35	0.16	0.44	**0.60**	0.20	**0.29**	0.28	-0.09	-0.02	-0.03	-0.04
35%	**0.50**	0.39	**0.58**	0.48	**0.54**	**0.21**	0.09	-0.06	0.25	-0.03	0.02	-0.12
Mean	0.45	0.43	0.42	0.40	0.38	0.31	0.18	0.06	0.05	0.02	-0.06	-0.15
Stress^b^	0.176	0.112	0.205	0.112	0.145	0.296	0.312	0.059	0.265	0.045	0.001	0.106

^a^ Datasets for which the ordination was estimated using hybrid multidimensional scaling (because it performed better). Non-metric multidimensional scaling was used in ordinate the other datasets.

^b^ Stress quantifies the badness of fit for each ordination [8].

Two factors–taxon and grid cell size–were associated with SAI. Although comparisons among positive SAI values are meaningful (SAI of 0.50 is twice as good as 0.25), means, correlation coefficients, and t-tests involving negative SAI values should be interpreted with caution (SAI of -0.50 is not necessarily twice as bad as -0.25, nor is it the mirror image of +0.50). Because 2 of our 12 SAI values were negative, the p-values should be treated as approximate. SAI values were higher for bird datasets (mean = 0.42, SD = 0.03, n = 3) than for plant datasets (mean = 0.15, SD = 0.22, n = 8; 2-tailed t-test with unequal variances, *P* = 0.01). Across the 7 atlas datasets, β diversity was a better surrogate for datasets with 10-km to 14-km grid cells (mean = 0.31, SD = 0.18, n = 4) than for atlas datasets with 25-km grid cells (mean = 0.01, SD = 0.06, n = 3; 2-tailed t-test with unequal variances, *P* = 0.04). None of the other 4 factors influenced SAI in a coherent way. SAI for datasets based on inventory data (mean = 0.24, SD = 0.25, n = 5) were not significantly different than SAI for datasets based on atlas data (mean = 0.18, SD = 0.23, n = 7, 2-tailed t-test with equal variances, *P* = 0.64). In tests of correlation (12 cases in each test), SAI did not co-vary with spatial extent (r = -0.19, *P* = 0.55), stress value of the ordination (r = +0.35, *P* = 0.27), or number of species (r = +0.24, *P* = 0.45). Inspection of scatterplots suggested only one potential nonlinear influence on SAI: the 5 highest SAI values were associated with stress values between 0.11 and 0.20 and SAI decreased at higher or lower stress values. Most surprisingly, the 4 datasets with stress values < 0.11 were associated with low SAI values (-0.15 to +0.06). Ordination plots for each of these 4 datasets (plants of Botswana, Namibia, Zimbabwe, and Sequoia-Kings Canyon National Park) had apparent outliers. The dataset for Irish mammals also had apparent outliers; the other 7 datasets did not. Deleting suspected outliers (4 to 44 outliers per dataset) resulted in a more “normal” ordination plot for each of these 5 datasets, but did not affect stress values in any consistent way, and did not improve SAI values.

## Discussion

For 6 of the 12 datasets we used, sites selected to maximize β diversity represented species about 30% to 45% as efficiently as direct selection of sites, meaning β diversity was 30% to 45% as effective as having full knowledge of species locations in its ability to improve on random selection of sites. Efficiency was consistent regardless of the fraction of the landscape prioritized. Across the 7 atlas datasets, β diversity was a better surrogate at cell sizes of 100–200 km^2^ than at cell sizes of 625 km^2^, supporting the idea [6] that β diversity is a better surrogate at finer resolution.

We are aware of only one other selection-based test of β diversity. In that study, Araujo et al. [6] reported that performance of β diversity was statistically better than random (*P* < 0.05) for plants and reptiles, but not significantly better than random for birds and mammals in 50x50-km cells of Western Europe (SAI was not reported). These results are more pessimistic than ours, which may be partly due to use of a coarser cell size (50x50 km) than in any of our data sets, or due to other statistical issues [5].

β diversity was a good surrogate for all 3 bird datasets, but a poor surrogate for the single mammal dataset (mammals of Ireland). Over 75% of Ireland is in agricultural land use, and only about 14% of its land area is forest and semi-natural land covers, compared to about 45% in the rest of Europe [23]. Perhaps human-caused changes in land cover and land use have relegated some mammals to remnant sites such that mammals in Ireland no longer have unimodal distributions in environmental space. It is not clear why β diversity was a good surrogate for 3 plant datasets and a poor surrogate for 4 plant datasets. It seems unlikely that plant assemblages reflect environmental conditions more closely in Chiapas, United Kingdom, and Shenandoah NP than in Zimbabwe, Namibia, Botswana, and Sequoya-Kings Canyon NP.

We detected no influence of type of biotic data (inventory versus atlas), number of species, stress value of the ordination, or spatial extent. In all cases, limited sample size (12 datasets) may have provided low statistical power. Our power to document an influence of spatial extent may have been further limited because we avoided using datasets with large (continental) spatial extents.

Surprisingly, stress values, which indicate badness of fit of an ordination [8], were not associated with SAI; indeed there was a non-significant *increase* in SAI with stress. The rule of thumb proposed by Clarke [8] suggested that stress values < 0.1 have “no real risk of false inferences” whereas stress values between 0.11 and 0.20 can be used with caution but are “potentially misleading.” But our 4 datasets with stress values < 0.11 (plants of Botswana, Namibia, Zimbabwe, and Sequoia-Kings Canyon National Park) produced 4 of the 5 worst SAI values, whereas stress values 0.11–0.20 were associated with the best SAI values. Although ordination plots for the 5 datasets with the lowest SAI values tended to have outliers, removing these outliers improved the ordination plots but not the SAI values. Further work using a larger number of datasets may better elucidate the characteristics of datasets that perform well or poorly.

Our evaluation of β diversity was optimistic because we evaluated surrogacy for the same taxon used to define ordination space. An obvious next step would be to test cross-taxon surrogacy in the β diversity framework. Such a test would require incidence data on 2 or more taxa at a common set of sites; if atlas data are used, our results suggest grid cells should be <625 km^2^. We found only one test of cross-taxon surrogacy in the β diversity framework; in that study Araujo et al. [6] found that cross-taxon efficiency was lower (> 10% decrease in number of species represented) than within-taxon efficiency in 5 of 12 comparisons, about the same (± 4%) in 5 comparisons, and higher (> 10% increase in species represented) in 2 comparisons.

Magurran & McGill [7] (page 292) concluded that almost all biodiversity metrics, including β diversity, “are basically descriptive in nature, and if they tie to any process at all it is a grossly oversimplified null process such as Poisson spatial randomness.” But recent analyses of spatial pattern demonstrate that β diversity is related to environmental gradients [24–26] and is congruent across broad taxonomic groups [26–29]. These papers have argued that their evidence supports the use of β diversity in conservation planning. However, such arguments eventually must be tested by selecting sites to represent β diversity and evaluating how well those sites represent species (or other conservation targets) [1, 30], as we have done here. Our results suggest that β diversity may be more than a mere descriptor of how species assemblages vary across the landscape. In particular, it appears that β diversity can be a useful metric to identify sites that maximize complementarity of sites prioritized for conservation.

We caution that support for β diversity as a within-taxon surrogate is a necessary but not sufficient condition for β diversity to serve as a good surrogate for conservation planning. Even if β diversity is a good within-taxon surrogate, it could still fail as a cross-taxon surrogate if different taxa respond to different environmental gradients. Although much work remains to be done to evaluate β diversity as a surrogate in conservation planning, our findings provide a strong reason to engage in that work. Because species distributions are so poorly known in most planning areas, conservation planners need reliable surrogates.
